# Comparative transcriptome analysis of inner blood-retinal barrier and blood–brain barrier in rats

**DOI:** 10.1038/s41598-021-91584-7

**Published:** 2021-06-09

**Authors:** Y. Li, A. Faiz, H. Moshage, R. Schubert, L. Schilling, J. A. Kamps

**Affiliations:** 1 Division of Neurosurgical Research, Medical Faculty Mannheim, Heidelberg University, Mannheim, Germany; 2 Department of Pathology and Medical Biology, Medical Biology Section, University Medical Center Groningen, University of Groningen, Groningen, The Netherlands; 3 Department of Gastroenterology and Hepatology, University Medical Center Groningen, University of Groningen, Groningen, The Netherlands; 4 European Center of Angioscience (ECAS), Medical Faculty Mannheim, Heidelberg University, Mannheim, Germany; 5 Physiology, Institute of Theoretical Medicine, Medical Faculty, Augsburg University, Augsburg, Germany

**Keywords:** Gene expression, Molecular medicine

## Abstract

Although retinal microvessels (RMVs) and brain microvessels (BMVs) are closely related in their developmental and share similar blood-neural barriers, studies have reported markedly different responses to stressors such as diabetes. Therefore, we hypothesized that RMVs and BMVs will display substantial differences in gene expression levels even though they are of the same embryological origin. In this study, both RMVs and BMVs were mechanically isolated from rats. Full retinal and brain tissue samples (RT, BT) were collected for comparisons. Total RNA extracted from these four groups were processed on Affymetrix rat 2.0 microarray Chips. The transcriptional profiles of these tissues were then analyzed. In the present paper we looked at differentially expressed genes (DEGs) in RMVs (against RT) and BMVs (against BT) using a rather conservative threshold value of ≥  ± twofold change and a false discovery rate corrected for multiple comparisons (*p* < 0.05). In RMVs a total of 1559 DEGs were found, of which 1004 genes were higher expressed in RMVs than in RT. Moreover, 4244 DEGs between BMVs and BT were identified, of which 1956 genes were ≥ twofold enriched in BMVs. Using these DEGs, we comprehensively analyzed the actual expression levels and highlighted their involvement in critical functional structures in RMVs and BMVs, such as junctional complex, transporters and signaling pathways. Our work provides for the first time the transcriptional profiles of rat RMVs and BMVs. These results may help to understand why retina and brain microvasculature show different susceptibilities to stressors, and they might even provide new insight for pharmacological interventions.

## Introduction

Organ function heavily depends on sufficient nutrient supply and waste removal occurring at the level of the microcirculation, basically the capillaries. Hence, the pathways of transendothelial solute exchange largely characterize the microcirculation in a given organ. The most complex design of microvessels (MVs) is present in the brain and in the retina, the latter derived from midbrain structures at an early stage of ontogenetic development. In these organs, highly specialized barriers termed blood–brain barrier (BBB) and inner blood-retina barrier (iBRB) separate the intra- and extravascular spaces. The most intriguing features of these barriers include (1) tight intercellular junctions to effectively prevent paracellular exchange of hydrophilic compounds, (2) a paucity of vesicular activity to minimize unselective transport, and (3) expression of a wide bunch of ion channels and transport systems including ATPases, symporters and antiporters, and carriers to enable selective transendothelial movement of solutes (for recent reviews, see Abbott et al.^1^ and Diaz-Coranguez and coworkers^2^).

Despite the close ontogenetic relationship between brain and retina and the common vascular supply there are remarkable differences between the retina MV (RMV) and brain MV (BMV) compartments, for instance in the response to systemic challenges such as chronic hyperglycemia in diabetes, arterial hypertension, and in the retinopathy of prematurity model with early postnatal hypoxia-hyperoxia challenges^3–5^. In each of these conditions, the RMVs are much more prone to failure than the BMVs, and subsequently, organ function is more seriously impaired in the retina than in the brain. Moreover, there is evidence to suggest that the retina and brain microcirculation may behave differently even under physiological conditions. This is exemplified by significantly different activity levels of the p-glycoprotein transporter (gene symbol, ABCB1), a member of the ATP-binding cassette (ABC) efflux transporter family^6^ and of high and low affinity neurotransmitter / organic acid transporters^7^. Furthermore, indirect evidence comes from studies addressing the level of expression/activity in isolated MVs or purified endothelial cells compared with the respective full tissue^8–13^.

Although the BBB and iBRB are prominent features of the RMV and BMV endothelial cells, the microvascular structure and function in the retina and brain require close interaction with the second type of mural cells, the pericytes^14^. Pericytes surround the abluminal side of the capillary wall and contribute to microvascular development, network stabilization and remodeling, and blood flow regulation^15–17^. In fact, the ratio of endothelial cells to pericytes is suspiciously high in the retina and brain^13^, pointing to their prominent role in RMVs and BMVs physiology within the neurovascular unit^14^. Therefore, to fully characterize the blood-neuronal barrier properties, isolated RMVs and BMVs consisting of endothelial cells and pericytes are in many respects superior to the use of individual cell types such as isolated endothelial cells or pericytes. Therefore, we have previously developed a method, which allows high yield high purity isolation of the microvascular compartment from rat brain and retina. We characterized these isolates histologically and by semi-quantitative real time polymerase chain reaction (qRT-PCR) methodology which showed significant enrichment of markers of microvascular-specific cells and a distinct depletion of parenchymal cell markers (covering neuronal, astrocytic, and photoreceptor cells) compared to full tissues^13^. Based on these results we have now performed a transcriptome-wide analysis to systematically compare RMVs and BMVs obtained from the same group of rats. We hypothesize that these two types of MVs will display substantial differences in gene expression levels even though they are of the same embryological origin. This heterogeneity at the transcriptome level may translate into differences in physiological responses, and it may also play a role in the differential susceptibility of the iBRB and BBB to stressors like high blood glucose.

## Results

**Figure 1:**
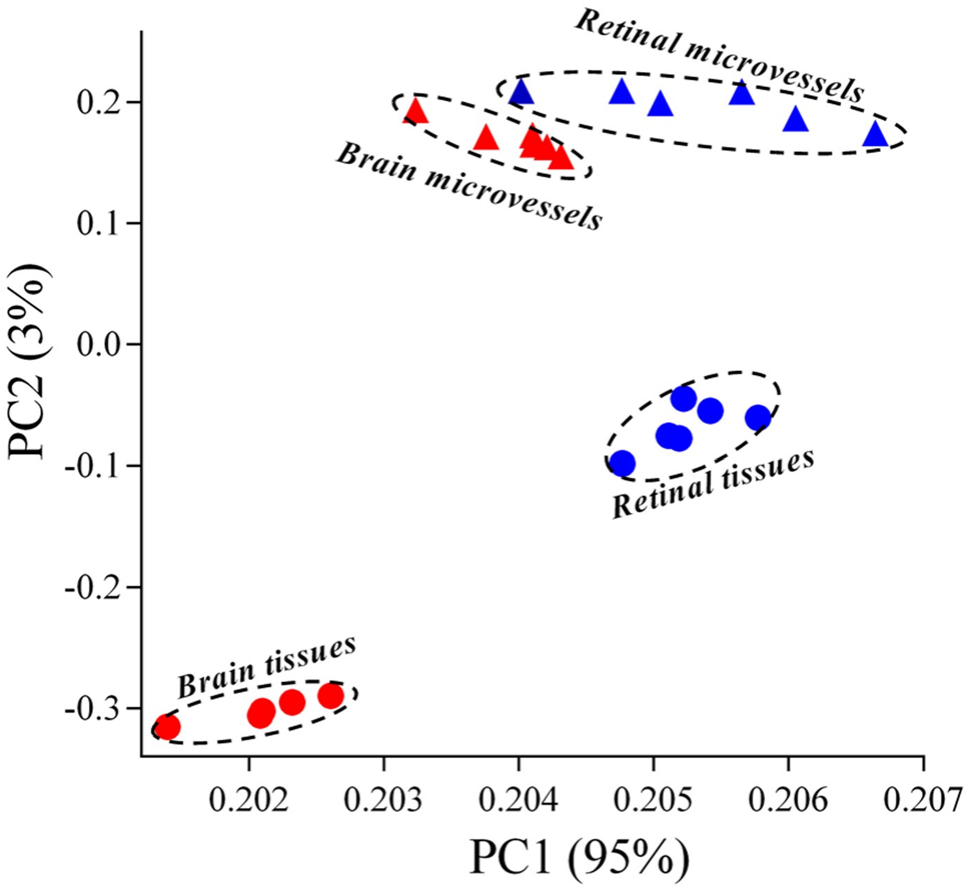
Principal component analysis (PCA) performed on whole transcriptome genes identified from RMVs, BMVs, RT and BT of rats. The first component (PC1) with a variance of 95% is on the X-axis and the second component (PC2) with a variance of 3% is on the Y-axis. Each dot represents one tissue sample. RMVs, retinal microvessels; BMVs, brain microvessels; RT, retinal tissue; BT, brain tissue.

A total of 20,743 genes were measured using Affymetrix rat 2.0 microarrays for transcriptome analysis of RMVs and BMVs and full retina tissue (RT) and brain tissue (BT). The gene expression data were at first analyzed by principal components analysis (PCA) which revealed 4 clusters with wide separation of RT and BT samples and somewhat less but still clear distinction of the RMVs and BMVs samples (Fig. 1). The principal components 1 and 2 accounted for 95% and 3% of the variations, respectively, showing a high degree of homogeneity within the different tissue samples.

### Overall transcriptional comparison between RMVs and RT

**Figure 2:**
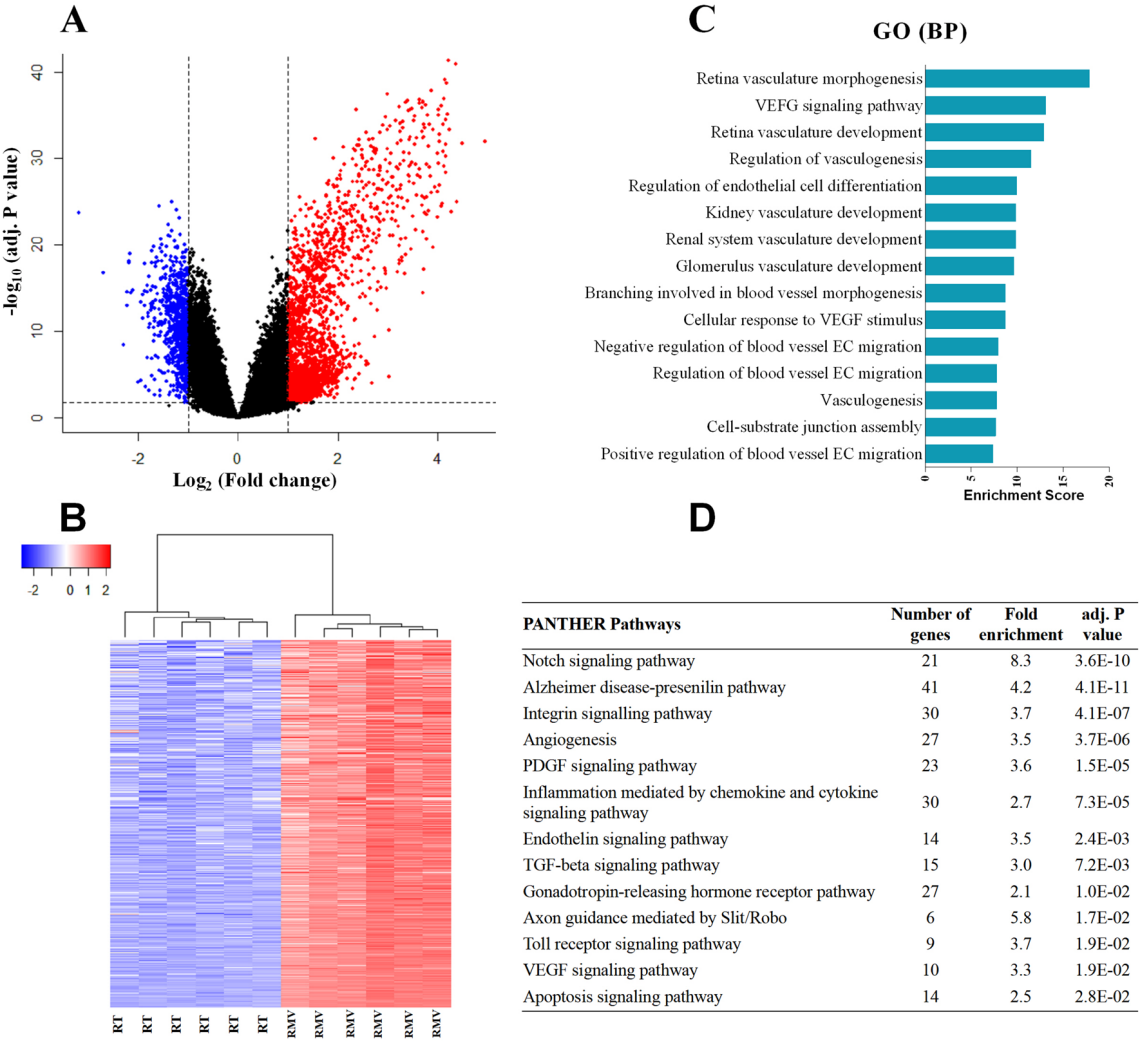
Gene expression comparison between retinal microvessels (RMVs) and retinal tissue (RT) in rats (n = 6). **(A)** Volcano plot for the RMVs versus RT whole transcriptomes. The red dots indicate the genes that are significantly higher expressed (fold change [FC] ≥ 2.0 and adjusted p < 0.05) in RMVs compared to RT, while blue dots indicate the lower expressed genes (FC < -2.0 and adjusted p < 0.05). **(B)** Hierarchical cluster analysis for genes that are significantly enriched (FC > 2.0 and adjusted p < 0.05) in RMVs. **(C,D)** Top 15 gene ontology (GO) biological processes (BP) and top 12 PANTHER pathways identified from the RMVs enriched genes. All the biological processes shown are ranked by enrichment score with a Bonferroni adjusted p < 0.05.

In the transcriptional comparison between RMVs and RT we identified 1,559 differentially expressed genes (DEGs, defined by fold change [FC] ≥  ± 2.0 and false discovery rate [FDR] adjusted p < 0.05), of which 1,004 genes were higher expressed, while 555 genes were lower expressed in RMVs than in RT (Fig. 2A). Hierarchical cluster analysis of the 1,004 genes significantly enriched in the RMVs showed a clear separation between the groups (Fig. 2B). To investigate the functional classifications of those RMVs enriched genes, gene ontology (GO) biological process categories were analyzed. The top 15 GO biological processes ranked by enrichment score (Fig. 2C) represent mainly vasculature-related biological processes including “retina vasculature morphogenesis” and “retina vasculature development”. Further investigation of the enriched pathways employing the PANTHER database identified 13 pathways showing a ≥ twofold enrichment in RMVs. Again, many of these pathways related to microvasculature features e.g., “Notch signaling pathway”, “Toll receptor signaling pathway” and “Angiogenesis” (Fig. 2D).

### Overall transcriptional comparison between BMVs and BT

**Figure 3:**
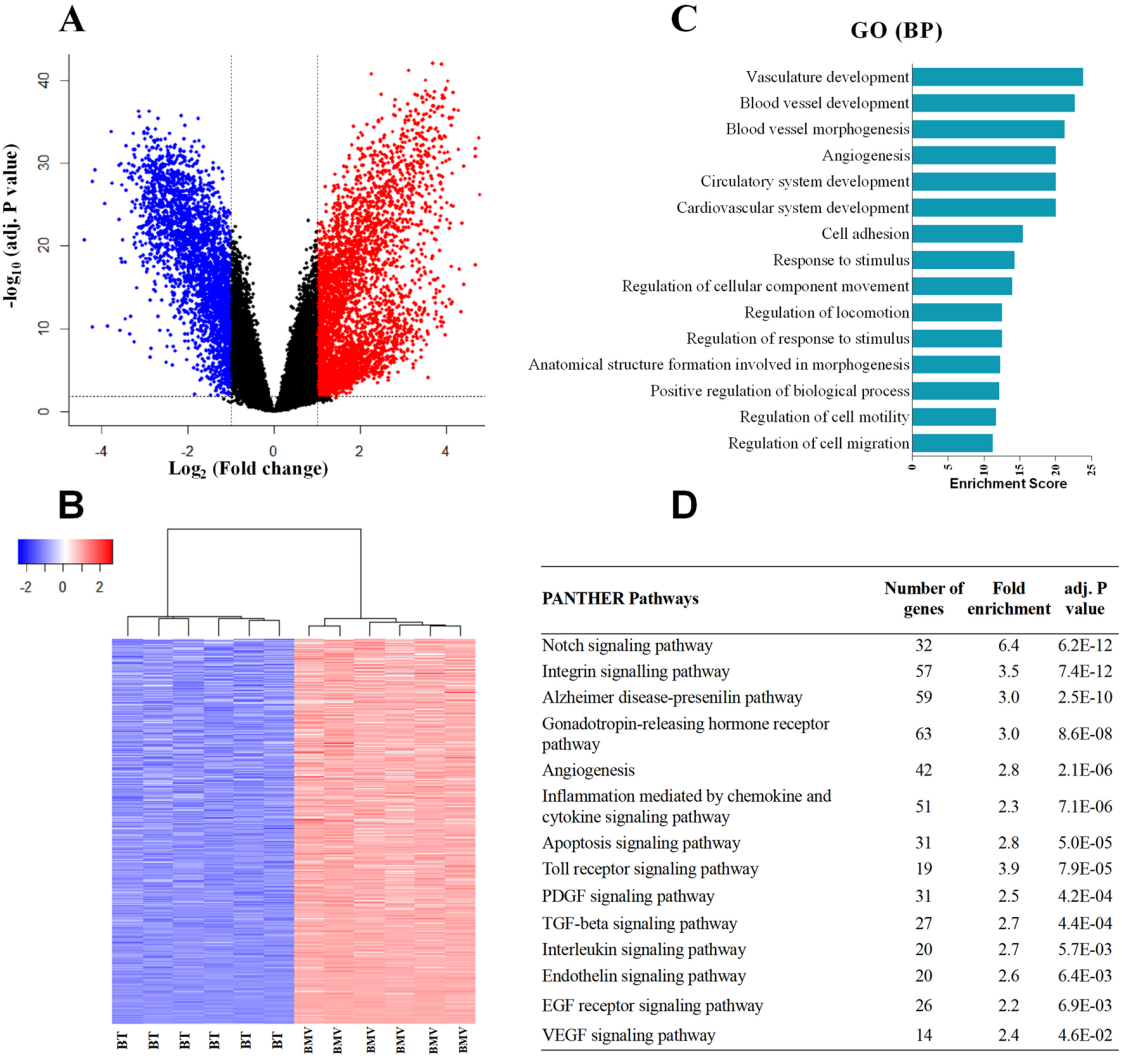
Gene expression comparison between brain microvessels (BMVs) and brain tissue (BT) in rats (n = 6). **(A)** Volcano plot for the BMVs versus BT whole transcriptomes. The red dots indicate the genes that are significantly higher expressed (fold change [FC] ≥ 2.0 and adjusted p < 0.05) in BMVs compared to BT, while blue dots indicate the lower expressed genes (FC ≤ -2.0 and adjusted p < 0.05) in BMVs. **(B)** Hierarchical cluster analysis for genes that are significantly enriched (FC > 2.0 and adjusted p < 0.05) in BMVs. **(C,D)** Top 15 gene ontology (GO) biological processes (BP) and 12 PANTHER pathways identified from the BMVs enriched genes. All the biological processes shown are ranked by enrichment score with a Bonferroni adjusted p < 0.05.

Transcriptional comparison between BMVs and BT revealed 4,244 DEGs with 1956 genes higher and 2,288 genes lower expressed in BMVs than in BT (Fig. 3A). Hierarchical cluster analysis of the 1,956 genes significantly enriched in the BMVs showed clear separation between the groups (Fig. 3B). Similar to the findings in RMVs, functional GO-based classification of the BMVs enriched genes contained many vasculature-related categories among the top 15 hits ranked by enrichment score (Fig. 3C), although the categories differed somewhat from those in RMVs. In contrast, applying the PANTHER database analysis onto BMVs (Fig. 3D) revealed a considerable agreement of vasculature-related enriched pathways with RMVs as reflected by “Notch signaling pathway”, “Toll receptor signaling pathway” and “Angiogenesis” also being among the most highly ranked.

### Validation of target genes showing significant enrichment in RMVs and BMVs

**Figure 4:**
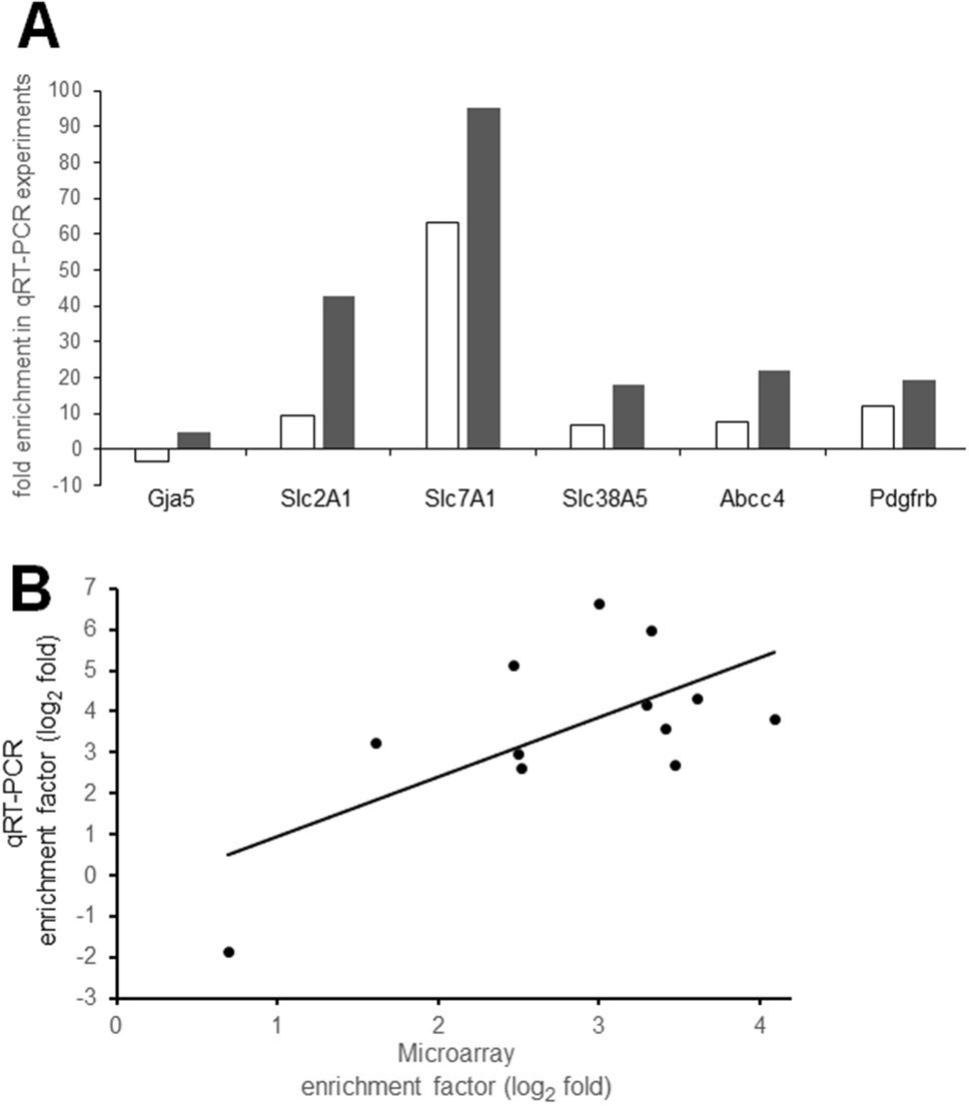
Enrichment of 6 target genes in retina microvessels (RMVs) and brain microvessels (BMVs) over the respective full tissues. **(A)** Fold changes obtained in the semiquantitative RT-PCR (qRT-PCR) measurements in RMVs (open bars) and BMVs (filled bars). Gja5, gap junction alpha-5 protein (also termed Cx40); Slc2A1, solute carrier family 2 member 1; Slc7A1, solute carrier family 7 member 1; Slc38A5, solute carrier family 38 member 5; Abcc4, ATP binding cassette subfamily C member 4; Pdgfrb, platelet derived growth factor receptor beta. **(B)** Agreement of results obtained by microarray measurements (abscissa) and qRT-PCR measurements (ordinate). In this graph enrichment is indicated as log_2_ fold changes. The regression line and the corresponding correlation coefficient show the significant agreement of data obtained by the two methods.

For validation of the microarray data qRT-PCR measurements were performed for 6 target genes and 4 reference genes (listed in Table 1). The factors of enrichment in RMVs and BMVs over the respective tissue samples (expressed as log_2_ fold values) displayed a significant agreement of the data obtained by microarray and qRT-PCR (Pearson correlation: r = 0.644, n = 12, p = 0.02) (Fig. 4B). Moreover, enrichment of 5 endothelial cell marker genes (endothelial nitric oxide synthetase [NOS3], TEK tyrosin-kinase [Tie2], von Willebrand factor [vWF], claudin 5 [Cld5], solute carrier (SLC) 2,1a [Slc2a1, previously termed Glut-1]) and 3 pericyte markers (platelet-derived growth factor receptor, beta polypeptide [PDGFRb], chondroitin sulfate proteoglycan 4 [CSPG4], and regulator of G-protein signaling 5 [RGS5]) taken from our previous study^13^ also displayed a significant correlation with the respective microarray data (Spearman rank test: r = 0.597, n = 16, p < 0.02). These observations strongly suggest good suitability and reliability of our microarray data for comparative gene expression analysis in the microvascular compartments.

**Table 1:** Features of the assays used for semiquantitative real time polymerase chain reaction (qRT-PCR) for validation and conformation of microarray measurements.

	Gene symbol	Accession number	Assay ID	Assay location	Amplicon length
*Reference genes*					
Rattus norvegicus beta-2 microglobulin	B2m	NM_012512	Rn00560865_m1	86	58
Rattus norvegicus actin, beta	Actb	NM_031144	Rn00667869_m1	881	91
Rattus norvegicus glyceraldehyde-3-phosphate dehydrogenase	Gapdh	NM_017008	Rn01775763_g1	1153	174
Rattus norvegicus hypoxanthine phosphoribosyltransferase 1	Hprt1	NM_012583	Rn01527840_m1	673	64
*Target genes*					
Rattus norvegicus gap junction protein alpha 5	Gja5	NM_019280	Rn00570632_m1	60	83
Rattus norvegicus solute carrier family 2 member 1	Slc2a1	NM_138827.1	Rn01417099_m1	1281	73
Rattus norvegicus solute carrier family 7 member 1	Slc7a1	NM_138827.1	Rn00565399_m1	961	76
Rattus norvegicus solute carrier family 38, member 5	Slc38a5	NM_138854.1	Rn00684896_m1	610	59
Rattus norvegicus ATP binding cassette subfamily C member 4	Abcc4	NM_133411.1	Rn01465702_m1	914	64
Rattus norvegicus platelet derived growth factor receptor beta	Pdgfrb	NM_031525	Rn00709573_m1	726	53

### Overall transcriptional comparison between the RMVs and BMVs

**Figure 5:**
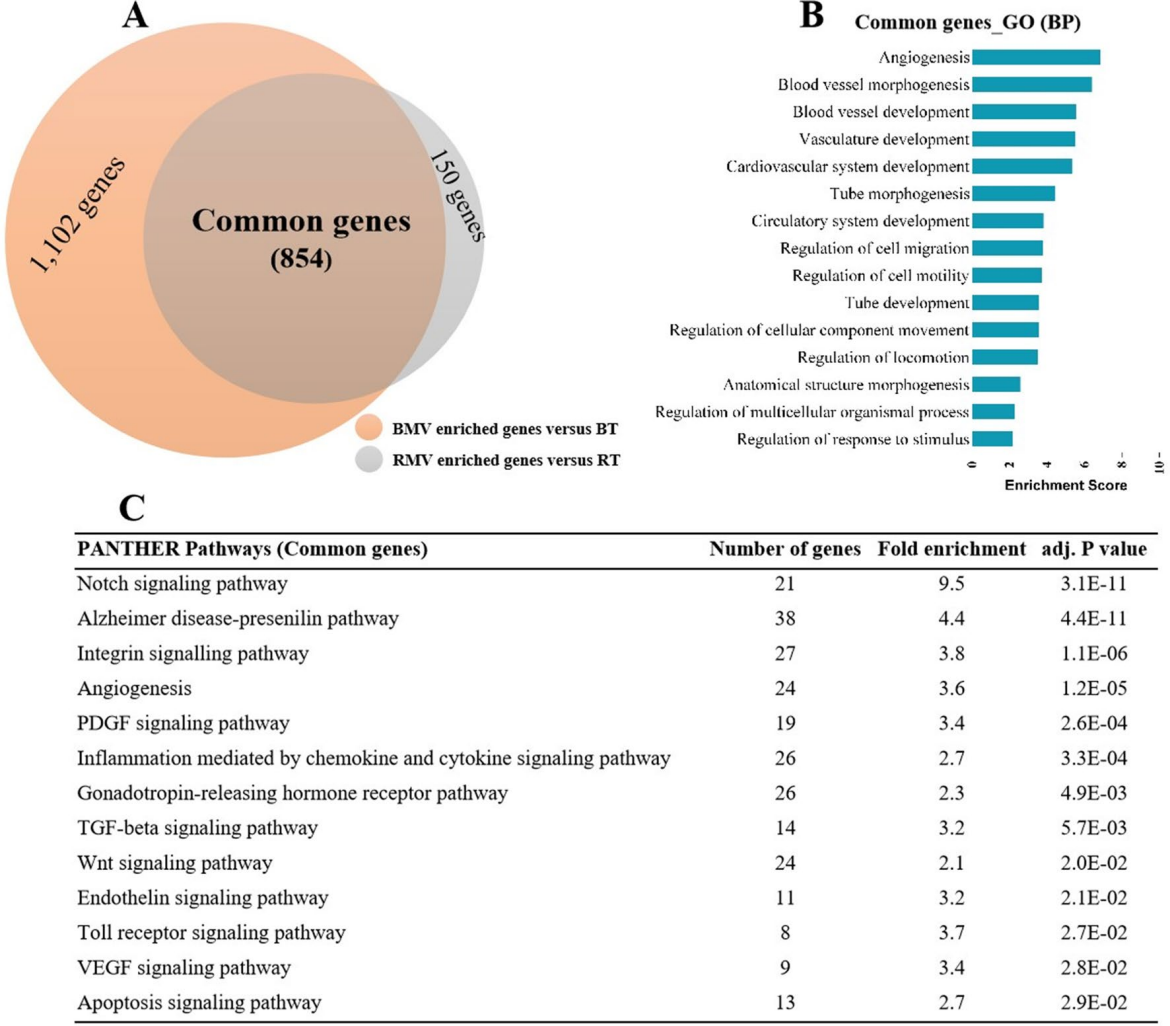
Gene expression comparison between RMVs and BMVs in rats (n = 6). **(A)** Venn diagram showing the numbers of genes that are significantly higher expressed in microvessels (MVs) obtained from the retina (RMVs, 1,004 genes) and from the brain (BMVs, 1956 genes). A total of 854 genes were commonly enriched in both, RMVs and BMVs. **(B)** Top 15 gene ontology (GO) biology processes (BP) identified from the 854 common genes. **(C)** Top 13 PANTHER pathways identified from the 854 common genes. All the biological processes shown are ranked by enrichment score with a Bonferroni adjusted p < 0.05 level of significance.

Based on the overlap between the genes > twofold enriched in RMVs and BMVs, we identified 854 common genes (accounting for 85.1% of RMVs and 43.7% of BMV genes, respectively) (Fig. 5A). Within the group of common genes, vasculature-related biological processes and functional pathways showed the highest enrichment scores using the gene ontology- and PANTHER-based classifications, respectively (Fig. 5B, C). For obvious reasons these results largely repeat those found for RMVs and BMVs compared to the respective full tissue samples thus further underlining the similarities between both microvascular compartments.

### Expression pattern of junctional complex genes in RMVs and BMVs

To further characterize the gene expression profiles of RMVs and BMVs, we highlighted 3 gene groups including (1) junctional complex formation, (2) endothelial transporters, and (3) endothelial / pericyte signaling pathways. Nine genes encoding for junctional complex proteins displayed a ≥ twofold enrichment in at least one MV compartment over the respective tissue (Tab.le 2). Of these 9 genes, 3 showed a significantly lower expression level in RMVs than in BMVs: occludin (Ocln), connexin (Cx) 43, and Cx40. Most notably, one gene, Cx43 even showed a considerably lower expression level in BMVs than in BT yielding a fold enrichment of −1.5-fold.

**Table 2:** List of intercellular junction genes differentially expressed in rat retina and/or brain microvessels vs. retina and brain tissue samples.

Symbol	RMVs	RT	RMVs versus RT		BMVs	BT	BMVs versus BT		RMVs versus BMVs	
			*FC*	*adj. P*			*FC*	*adj. P*	*FC*	*adj. P*
Ocln	9.7 ± 0.17	6.4 ± 0.13	9.8	2E−27	10.8 ± 0.04	7.4 ± 0.06	10.7	6E−29	− 2.1	8E−10
Cldn5	13.1 ± 0.1	9.4 ± 0.13	12.1	7E−34	13.4 ± 0.01	10.3 ± 0.04	8.4	3E−31	− 1.2	2E−02
Jam2	12 ± 0.05	10.9 ± 0.02	2.1	5E−20	12.2 ± 0.04	10.2 ± 0.02	4.0	8E−32	− 1.1	8E−03
Cdh5	11.4 ± 0.1	7.1 ± 0.06	21.1	2E−37	12 ± 0.01	8.1 ± 0.11	15.6	8E−36	− 1.5	1E−05
ZO-1	11.6 ± 0.09	10.8 ± 0.03	1.7	3E−15	12.2 ± 0.03	10.6 ± 0.03	3.1	3E−28	− 1.5	4E−12
Ctnna1	8.6 ± 0.12	8.1 ± 0.07	1.4	1E−05	9.3 ± 0.01	8.1 ± 0.03	2.3	3E−16	− 1.6	9E−08
Cx37	10.1 ± 0.07	6.9 ± 0.07	9.2	6E−27	10.8 ± 0.08	6.9 ± 0.10	14.8	4E−31	− 1.6	2E−05
Cx43	9.0 ± 0.10	7.4 ± 0.36	3.0	2E−08	10.9 ± 0.06	11.5 ± 0.02	− 1.5	5E−02	− 3.7	5E−09
Cx40	6.9 ± 0.05	6.2 ± 0.09	1.6	5E−06	8.6 ± 0.09	6.1 ± 0.13	5.7	2E−25	− 3.2	7E−18

Results are mean ± SEM of log_2_ transformed microarray intensity values (n = 6). RMVs, retinal microvessels; RT, retinal tissue; BMVs, brain microvessels; BT, brain tissue; *FC*, fold change; *adj. P*, false discovery rate adjusted p value; Ocln, occluding; Cldn5, claudin 5; Jam2, junctional adhesion molecule 2; Cdh5, VE-cadherin; ZO-1, zonula occludens protein 1; Ctnna1, catenin α1; Cx, connexin.

### Expression pattern of membrane carrier and transporter genes in RMVs and BMVs

We identified 355 SLC and 47 ATP-binding cassette (ABC) transporter genes in the arrays. Among the SLCs, 56 genes showed a more than twofold enrichment in at least one MV compartment (Table 3). Moreover, among the ABCs a total of 8 genes showed a more than twofold enrichment in either RMVs or BMVs (Table 4). Interestingly all genes that did not surmount the threshold of enrichment were found in RMVs. In accord, almost half the carrier and transporter genes listed in Tables 3 and 4 showed marked differences between RMVs and BMVs pointing to considerably distinct patterns of gene expression in both microvascular compartments. Most notably, Slc16a (monocarboxylate transporter-1) showed a higher expression level in RMVs than in BMVs.

**Table 3:** List of solute carrier genes differentially expressed in rat retina and/or brain microvessels vs. retina and brain tissue samples.

Symbol	RMVs	RT	RMVs. vs. RT		BMVs	BT	BMVs. vs. BT		RMVs. vs. BMVs	
			*FC*	*adj. P*			*FC*	*adj. P*	*FC*	*adj. P*
Slc2a1	13.4 ± 0.05	11.8 ± 0.03	3.0	3E-22	13.5 ± 0.03	11.1 ± 0.03	5.5	5E-31	-1.1	2E-01
Slc2a4	7.4 ± 0.08	6.3 ± 0.07	2.1	1E-10	8.4 ± 0.07	6.2 ± 0.13	4.5	1E-22	-2.0	7E-10
Slc2a12	7.5 ± 0.13	6.7 ± 0.13	1.7	1E-04	10.2 ± 0.04	7.1 ± 0.1	8.7	3E-23	-6.4	1E-19
Slc3a2	11.1 ± 0.07	10.6 ± 0.06	1.3	5E-05	11.6 ± 0.04	10.3 ± 0.04	2.5	5E-20	-1.4	2E-06
Slc5a5	6.8 ± 0.11	6.4 ± 0.16	1.4	3E-02	8.6 ± 0.06	6.3 ± 0.07	4.9	9E-18	-3.5	3E-13
Slc6a20	6.2 ± 0.04	6.2 ± 0.28	1.0	1E + 00	10.1 ± 0.09	7.2 ± 0.15	7.8	2E-19	-15.2	6E-24
Slc7a1	11.9 ± 0.12	8.6 ± 0.2	10.0	9E-23	12.5 ± 0.07	9.5 ± 0.04	8.0	1E-21	-1.5	1E-02
Slc7a2	9 ± 0.07	8.2 ± 0.16	1.7	3E-06	10.8 ± 0.08	8.8 ± 0.06	3.9	1E-18	-3.5	2E-16
Slc7a5	11.8 ± 0.06	11 ± 0.07	1.7	7E-07	12.3 ± 0.04	9.7 ± 0.08	5.7	4E-25	-1.4	4E-03
Slc7a15	8.2 ± 0.13	6.3 ± 0.14	3.6	2E-16	8.6 ± 0.09	6.3 ± 0.07	5.0	4E-21	-1.3	2E-02
Slc8b1	6.9 ± 0.05	6 ± 0.05	1.8	1E-07	8 ± 0.08	6.6 ± 0.11	2.7	1E-14	-2.2	8E-11
Slc9a3r2	11 ± 0.08	8 ± 0.13	7.9	5E-31	11.7 ± 0.05	8.7 ± 0.06	7.7	3.E-31	-1.6	7E-07
Slc10a3	9.1 ± 0.12	7.5 ± 0.2	3.2	8E-08	9.8 ± 0.17	7.3 ± 0.24	5.4	7E-13	-1.5	4E-02
Slc10a7	9 ± 0.08	8.2 ± 0.09	1.7	2E-07	9 ± 0.05	7.2 ± 0.06	3.5	1E-19	-1.0	8E-01
Slc12a4	8.2 ± 0.18	7.4 ± 0.06	1.8	2E-07	9.4 ± 0.04	8 ± 0.09	2.6	9E-14	-2.3	2E-11
Slc12a7	9.5 ± 0.06	9 ± 0.05	1.4	1E-04	10.1 ± 0.08	7 ± 0.09	8.8	4E-33	-1.5	7E-07
Slc12a8	5.9 ± 0.08	5.8 ± 0.13	1.1	6E-01	6.7 ± 0.1	5.7 ± 0.06	2.1	5E-09	-1.7	4E-06
Slc13a4	6.5 ± 0.11	6.3 ± 0.1	1.1	5E-01	10.6 ± 0.09	7.7 ± 0.05	7.6	1E-23	-18.1	4E-30
Slc15a3	7.8 ± 0.06	6.2 ± 0.11	2.9	1E-11	8.8 ± 0.1	7.5 ± 0.05	2.5	5E-10	-2.0	5E-07
Slc16a1	11.4 ± 0.11	10.4 ± 0.05	2.1	1E-04	9.7 ± 0.18	8.7 ± 0.09	2.0	2E-04	3.4	4E-09
Slc16a2	8.6 ± 0.13	7.3 ± 0.09	2.4	6E-13	10.9 ± 0.03	8.5 ± 0.1	5.1	6E-24	-4.9	9E-23
Slc16a4	7.4 ± 0.15	4.8 ± 0.11	6.1	3E-20	8.4 ± 0.1	5.1 ± 0.07	9.9	2E-25	-2.0	1E-06
Slc16a11	6.3 ± 0.06	6.2 ± 0.05	1.0	9E-01	7.7 ± 0.06	6.3 ± 0.05	2.6	1E-13	-2.7	1E-13
Slc16a12	6 ± 0.17	5.2 ± 0.15	1.7	2E-03	7.9 ± 0.04	5.2 ± 0.17	6.8	1E-17	-3.9	1E-11
Slc16a13	7.1 ± 0.16	6.4 ± 0.09	1.6	2E-05	8.3 ± 0.05	6.9 ± 0.07	2.7	4E-14	-2.4	2E-11
Slc19a3	10.4 ± 0.16	6.7 ± 0.12	13.1	4E-20	11 ± 0.12	7.5 ± 0.16	11.2	1E-19	-1.5	3E-02
Slc22a5	8.7 ± 0.11	7 ± 0.14	3.4	1E-11	9.1 ± 0.14	7.1 ± 0.11	3.9	8E-14	-1.3	2E-01
Slc22a6	6.3 ± 0.08	6.2 ± 0.15	1.1	7E-01	10.2 ± 0.11	6.7 ± 0.05	11.5	2E-23	-14.9	7E-25
Slc22a8	9.7 ± 0.14	7 ± 0.28	6.3	2E-12	11.5 ± 0.08	7.8 ± 0.03	12.6	5E-18	-3.5	1E-07
Slc22a18	7.6 ± 0.07	6.2 ± 0.09	2.7	6E-12	8.5 ± 0.04	6 ± 0.13	5.9	3E-22	-1.9	1E-06
Slc25a2	7.6 ± 0.13	7.3 ± 0.13	1.2	4E-01	7.2 ± 0.1	5.9 ± 0.26	2.5	8E-08	1.3	1E-01
Slc25a35	10.7 ± 0.11	10.6 ± 0.05	1.1	6E-01	9.6 ± 0.06	8 ± 0.08	3.2	2E-19	2.1	5E-12
Slc25a45	7.2 ± 0.16	6 ± 0.15	2.3	4E-08	8.4 ± 0.1	6.1 ± 0.12	4.9	1E-17	-2.3	7E-08
Slc26a10	6.4 ± 0.07	5.9 ± 0.07	1.4	2E-04	9.2 ± 0.06	6.1 ± 0.06	8.8	6E-31	-7.1	1E-28
Slc28a3	6.8 ± 0.11	5.9 ± 0.08	1.9	1E-07	7.5 ± 0.16	5.8 ± 0.05	3.2	3E-16	-1.6	2E-05
Slc30a1	9.2 ± 0.11	7.7 ± 0.08	2.8	5E-15	10.2 ± 0.04	8.1 ± 0.06	4.1	6E-21	-2.0	2E-09
Slc30a10	7.4 ± 0.05	6.6 ± 0.08	1.8	4E-07	8.7 ± 0.06	7.3 ± 0.05	2.6	9E-14	-2.4	1E-11
Slc31a1	10.8 ± 0.09	9.8 ± 0.08	2.0	1E-11	11.1 ± 0.07	9.7 ± 0.02	2.6	6E-17	-1.3	1E-02
Slc31a2	8.9 ± 0.06	8.7 ± 0.05	1.2	6E-02	7.5 ± 0.04	6.3 ± 0.06	2.3	3E-15	2.7	4E-17
Slc35f2	9.3 ± 0.16	6.8 ± 0.13	5.5	7E-16	9.7 ± 0.2	7.1 ± 0.12	6.0	3E-17	-1.3	2E-01
Slc38a5	9.6 ± 0.19	6.1 ± 0.1	11.1	3E-18	10.4 ± 0.12	6.3 ± 0.16	17.0	6E-22	-1.8	5E-03
Slc38a11	6.6 ± 0.1	5.8 ± 0.07	1.7	3E-05	7.2 ± 0.07	5.8 ± 0.08	2.7	3E-12	-1.5	8E-04
Slc39a1	8.9 ± 0.05	8.1 ± 0.05	1.8	2E-09	9.3 ± 0.05	8.2 ± 0.09	2.1	3E-13	-1.3	4E-03
Slc39a4	6.5 ± 0.07	6.1 ± 0.15	1.3	5E-02	7.3 ± 0.08	5.8 ± 0.11	2.8	5E-14	-1.8	1E-06
Slc39a8	9.6 ± 0.11	6.6 ± 0.2	8.3	9E-20	10 ± 0.11	6.8 ± 0.22	9.7	8E-22	-1.3	1E-01
Slc39a10	11.5 ± 0.11	9.5 ± 0.06	4.0	5E-18	12.3 ± 0.06	10.9 ± 0.04	2.6	6E-13	-1.7	6E-06
Slc40a1	10.8 ± 0.22	6.8 ± 0.17	16.0	9E-22	11.8 ± 0.08	8 ± 0.09	13.9	2E-21	-2.0	3E-04
Slc43a1	6.9 ± 0.14	5.1 ± 0.11	3.6	2E-13	7.5 ± 0.09	5.5 ± 0.17	4.1	1E-15	-1.6	2E-03
Slc50a1	9.5 ± 0.02	8.5 ± 0.08	2.0	1E-15	10.2 ± 0.04	8.6 ± 0.04	2.9	3E-24	-1.5	3E-09
Slc52a2	8.5 ± 0.05	7.9 ± 0.06	1.5	2E-04	8.8 ± 0.07	6.9 ± 0.05	3.6	1E-17	-1.2	2E-01
Slc52a3	9.4 ± 0.14	6.3 ± 0.05	8.5	4E-26	10.7 ± 0.04	6.8 ± 0.05	15.5	2E-31	-2.5	6E-12
Slco1a2	11.4 ± 0.11	7.5 ± 0.13	14.6	2E-24	12.1 ± 0.02	8.6 ± 0.08	11.5	3E-23	-1.7	1E-03
Slco1c1	12.8 ± 0.11	9.5 ± 0.06	9.7	2E-17	13.3 ± 0.03	10.7 ± 0.04	5.9	5E-14	-1.5	8E-02
Slco2a1	8.1 ± 0.13	5.7 ± 0.08	5.5	5E-13	7.6 ± 0.14	5.6 ± 0.12	4.2	3E-11	1.4	1E-01
Slco2b1	9.2 ± 0.1	7.1 ± 0.08	4.6	4E-22	10.5 ± 0.01	8.1 ± 0.07	5.2	3E-24	-2.4	1E-12
Slco3a1	9.9 ± 0.05	8.3 ± 0.09	3.1	9E-21	10.3 ± 0.06	9.1 ± 0.1	2.3	2E-16	-1.4	2E-04

Results are mean ± SEM of log_2_ transformed microarray intensity values (n = 6). RMVs, retinal microvessels; RT, retinal tissue; BMVs, brain microvessels; BT, brain tissue; *FC*, fold change; *adj. P*, false discovery rate adjusted p value.SLC, solute carrier.

**Table 4:** List of ATP binding cassette transporter genes differentially expressed in rat retina and/or brain microvessels vs. retina and brain tissue samples.

Symbol	RMVs	RT	RMVs. vs. RT		BMVs	BT	BMVs. vs. BT		RMVs. vs. BMVs	
			*FC*	*adj. P*			*FC*	*adj. P*	*FC*	*adj. P*
Abcb1a	10.7 ± 0.14	7 ± 0.14	12.9	5E-23	11.6 ± 0.03	8 ± 0.07	12.3	2E-23	-1.9	6E-05
Abcc1	9.5 ± 0.09	8.8 ± 0.04	1.6	6E-05	8.7 ± 0.06	7.3 ± 0.08	2.7	2E-14	0.6	6E-06
Abcc3	7 ± 0.06	6.7 ± 0.04	1.2	3E-02	7.7 ± 0.04	6.3 ± 0.07	2.6	4E-18	-1.7	6E-09
Abcc4	9.2 ± 0.14	6.7 ± 0.08	5.6	9E-22	10.7 ± 0.08	7.1 ± 0.08	12.2	2E-29	-2.8	3E-13
Abcc6	6.6 ± 0.03	6 ± 0.07	1.5	3E-05	7.1 ± 0.06	5.7 ± 0.07	2.6	4E-16	-1.4	3E-04
Abcc9	11.4 ± 0.15	7.4 ± 0.13	16.3	7E-30	11 ± 0.08	7.5 ± 0.09	11.0	2E-27	0.7	1E-02
Abcd1	8.9 ± 0.1	7.3 ± 0.08	3.0	1E-16	9.6 ± 0.03	8.1 ± 0.12	2.8	1E-16	-1.6	2E-06
Abcg2	9.3 ± 0.14	5.4 ± 0.16	14.9	2E-18	10.5 ± 0.24	6.6 ± 0.22	14.4	7E-19	-2.2	4E-04
Abcg3l1	6 ± 0.09	5.2 ± 0.16	1.7	E-03	7.1 ± 0.12	5.3 ± 0.24	3.6	6E-11	-2.2	2E-05

Results are mean ± SEM of log_2_ transformed microarray intensity values (n = 6). RMVs, retinal microvessels; RT, retinal tissue; BMVs, brain microvessels; BT, brain tissue; *FC*, fold change; *adj. P*, false discovery rate adjusted p value. Abc, ATP binding cassette transporter.

### Expression pattern of signaling pathway genes in RMVs and BMVs

For the signaling between endothelial cells and pericytes, we found a couple of genes displaying a ≥ twofold enrichment in at least one of the MV compartments. These genes belong to the PDGFb / PDGFRb pathway, Tie2 / Angiopoietin (Ang) pathway, transforming growth factor beta (TGF-β) signaling pathway and Notch signaling pathway (Table 5). Compared with BMVs, the expression levels of Ang-1, TGFβ, and TGFβ receptor beta 3 (Tgfrb3) mRNA were significantly lower in RMVs (Table 5).

**Table 5:** List of signaling genes between endothelial cells and pericytes differentially expressed in rat retina and/or brain microvessels vs. retina and brain tissue samples.

Symbol	RMVs	RT	RMVs. versus RT		BMVs	BT	BMVs. versus BT		RMVs. versus BMVs	
			*FC*	*adj. P*			*FC*	*adj. P*	*FC*	*adj. P*
Pdgfb	9.3 ± 0.14	6.5 ± 0.05	7.0	7E−23	10 ± 0.06	7.2 ± 0.07	7.2	7E-24	−1.6	4E−05
Pdgfbrβ	11.4 ± 0.09	8 ± 0.08	10.6	4E−33	11.6 ± 0.06	8.3 ± 0.06	9.8	9E−33	−1.1	9E−02
Tie2	11.2 ± 0.11	7.5 ± 0.11	13.0	1E−33	12.1 ± 0.03	8.6 ± 0.06	11.0	3E−33	−1.9	1E−09
Ang-1	5.9 ± 0.12	5.5 ± 0.23	1.3	6E−02	7.3 ± 0.08	6.1 ± 0.1	2.2	3E−07	−2.6	2E−08
Ang-2	8 ± 0.13	6.9 ± 0.1	2.1	4E−07	8.6 ± 0.06	5.9 ± 0.16	6.6	1E−21	−1.5	2E−03
Tgfβ	8.6 ± 0.07	6.5 ± 0.06	4.3	3E−23	9.9 ± 0.04	6.9 ± 0.13	8.0	3E−30	−2.5	1E−14
Tgfbr2	9.5 ± 0.13	6.8 ± 0.07	6.5	6E−27	10.3 ± 0.05	7.3 ± 0.08	8.0	2E−29	−1.7	2E−07
Tgfbr3	10 ± 0.13	7.5 ± 0.09	5.7	6E−27	11.5 ± 0.04	8.6 ± 0.04	7.4	3E−30	−2.8	1E−17
Alk-1	10.1 ± 0.08	6.4 ± 0.08	13.0	1E−33	10.9 ± 0.03	6.9 ± 0.07	15.5	3E−35	−1.7	2E−07
Dll4	9.5 ± 0.09	6.4 ± 0.14	8.6	8E−20	10.2 ± 0.16	8.6 ± 0.06	10.1	8E−22	−1.6	3E−09
Jag-1	10.7 ± 0.04	9.8 ± 0.05	1.9	3E−11	10.9 ± 0.04	8.2 ± 0.05	10.0	2E−35	−1.1	8E−13
Notch1	10.5 ± 0.1	8.4 ± 0.08	4.3	1E−22	11.4 ± 0.05	6.9 ± 0.05	6.7	8E−29	−1.9	1E−10
Notch3	10.8 ± 0.18	7.4 ± 0.07	10.6	2E−27	11.5 ± 0.06	7.6 ± 0.12	13.0	3E−29	−1.6	2E−02

Results are mean ± SEM of log_2_ transformed microarray intensity values (n = 6). RMVs, retinal microvessels; RT, retinal tissue; BMVs, brain microvessels; BT, brain tissue; *FC*, fold change; *adj. P*, false discovery rate adjusted p value. Pdgfb, platelet derived growth factor beta; Pdgfbrβ. Pdgfb receptor β; Tie2, TEK tyrosin-kinase 2; Ang, angiopoietin; Tgfβ, transforming growth factor beta; Tgfbr, TGFβ receptor; Alk-1, activin receptor-like kinase 1; Dll4, delta like canonical Notch ligand 4; Jag-1, jagged canonical Notch ligand 1.

## Discussion

To our knowledge, this is the first study in which a detailed gene expression analysis was performed in isolated rat RMVs. Using a threshold of ≥ twofold enrichment in comparative transcriptome analysis we identified 1,004 genes enriched in RMVs over corresponding RT. Furthermore, we also comprehensively analyzed the whole transcriptional profile of BMVs and the respective BT. Significantly enriched genes were mainly related to vascular biological processes in both, RMV and BMV compartments. Our study also enables in-depth comparison of RMVs and BMVs, which is of major interest since embryologically the microvascular bed of the retina is deemed a derivative of the brain circulation. Despite this close ontogenetic relationship, the overall gene expression profiles of RMVs and BMVs displayed two distinct albeit closely neighbouring, clusters in the PCA analysis. Since both, RMVs and BMVs are characterized by a tight endothelial barrier and a suspiciously high endowment with pericytes, we scrutinized the classes of junctional complex, membrane transporter, and endothelial / pericyte signaling pathway genes.

Isolation of RMVs and BMVs was performed in exactly the same manner as described previously^13^. In this paper, we provided an in-depth characterization of the microvascular compartments showing that both, RMVs and BMVs were histologically devoid of any contamination by tissue fragments. Moreover, on the gene expression level, endothelial cell and pericyte marker genes displayed a distinct enrichment and a distinct depletion of astroglial, neuronal, and photoreceptor marker genes suggesting high purity of the isolated RMV and BMV compartments. This conclusion receives further support by the present study using microarray methodology. Again, each total RNA isolate underwent a strict quality control procedure using a chip-based electrophoretic method. Extracts were only used for further analysis if the RNA integrity number was at least 8 as recommended previously^18^. For bioinformatics analysis, a threshold value of an at least twofold enrichment was applied to identify DEGs. This value was chosen to limit the number of hits, although a somewhat lower enrichment factor, ≥ 1.5 fold has been suggested suitable for identifying significant DEGs when complex tissues are studied^19^.

In order to validate the enrichment factors obtained by the microarray measurements we used results from qRT-PCR. Firstly, in samples of total RNA from the tissue isolates produced in the present study the expression levels of 6 target genes and 4 reference genes were measured. The degree of enrichment, expressed as log_2_ fold changes in RMVs and BMVs displayed a good correlation with the respective values obtained by microarray analysis. Secondly, 8 target genes measured by microfluidic card methodology as reported in our previous paper^13^ also revealed a good agreement of the enrichment factors (again expressed as log_2_ fold changes in RMVs and BMVs) with the respective values obtained in the present microarray study. The panel of target genes selected in both approaches contains endothelial and pericyte marker genes with some overlap (Slc2A1 and PDGFRb). The consistency of results derived from a wide range of genes and different starting materials provides strong support to the reliability of our microarray data.

The RMVs and BMVs are characterized by the expression of tight barriers, termed iBRB and BBB. These are characterized by the formation of intercellular macromolecular complexes consisting of tight junctions, adherens junctions, and gap junctions between the endothelial cells^20^. Functionally, iBRB and BBB are similar, since the permeability surface product for the small molecular tracers mannitol and sucrose has been found largely comparable^21^. In our study, both RMVs and BMVs expressed high levels (≥ twofold enrichment in at least one MV compartment) of a number of junction-related genes. These results clearly emphasize the functional importance of the tight intercellular junctions, yet also suggest some differences in their make-ups in RMVs and BMVs. The overall enrichment of junctional complex genes in RMVs and BMVs is not necessarily conflicted by a markedly lower expression level of Cx43 in BMVs than in BT. In fact, there is a high level in BT of Cx43 which has previously been shown to be the predominant Cx subtype in adult brain tissue, abundantly expressed in astrocytes^22^.

In agreement with the present study, distinctly high expression levels of junctional complex genes have previously been found in rat cortical MVs, despite major differences in the methods used for vessel isolation and gene quantification^23^. The tightness of the iBRB and BBB, which has been related to the expression level of junctional genes, also determines the susceptibility to endogenous or exogenous insults. For example, Tien et al. found that the downregulation of Cx43 in the retina induced by Cx43 siRNA or diabetes following streptozotocin injection, contributed to compromised retinal vascular homeostasis^24^. More recently, using immunohistochemistry and vasomotor response assessment, Ivanova and coworkers reported that Cx43 gene expression level in rat retinal capillaries was significantly decreased by hyperglycemia and that this decrease contributed to the vasomotor decline of the inner retinal capillaries^25^. Furthermore, it has been demonstrated that in vitro downregulation of the Cx43/ZO-1 complex in RMV or BMV endothelial cells contributes to the breakdown of the iBRB and BBB^26,27^. In the present study, we demonstrate that in physiological conditions the expression levels of Cx43, Cx40, and Ocln were significantly lower in RMVs compared to BMVs while the difference for ZO-1 did not reach the predetermined DEG level. Based on these observations, we speculate that the lower baseline expression of many tight junction-associated genes in RMVs might contribute to a higher susceptibility to e.g. hyperglycemia-induced damage of the iBRB compared to the BBB.

The tight interendothelial junctions of the iBRB and BBB effectively block the paracellular passage of hydrophilic solutes. Therefore, these compounds depend on a transcellular route to enter (or leave) the parenchymal tissue. However, this cannot be simple diffusion (due to the lipid membranes covering the endothelial cells). In the light of the paucity of transport vesicles, the movement of bulk amounts of many solutes requires the presence of endothelial transporters, which, therefore, take center stage in the barrier function. Systematic studies of the transporter gene expression patterns in the rodent iBRB have been limited until recently due to the difficulty in isolating MVs of sufficient quality and purity. Instead of RMVs, retinal vascular endothelial cells and a conditionally immortalized retinal capillary endothelial cell line (TR-iBRB2) have been used as iBRB models. Using these models, Tachikawa et al.^9^ studying 19 in- and efflux transporter mRNAs identified 5 of these (Abca9, Abcb1b, Abcc3, Abcc4, Abcc6 and Abcg2) with a suspiciously high expression level. Other studies have shown that Slc7a1 (high affinity cationic amino acid transporter 1, [CAT1]), Slc7a5 (large neutral amino acids transporter small subunit 1 [LAT-1]), Slc16a1 (monocarboxylate transporter 1 [MCT-1]) and Slc22a5 (organic transport transporter 2 [OCTN2]) mRNA were expressed in the TR-iBRB2 cell line^28–31^. In our study employing whole transcriptome analysis, we identified 355 SLC transporter genes of which 56 genes were ≥ twofold enriched in at least one MV compartment. Moreover, we identified 8 out of 47 ABC transporter genes to display a ≥ twofold enrichment in at least one MV compartment. Our findings expand the database of transporter genes expressed in the iBRB and also increase our knowledge of similarities and differences between iBRB and BBB. For instance, we found that the expression level of Slc2A1, which is a major transporter of glucose across the barriers, was expressed at comparable levels in both RMVs and BMVs. In contrast, a previous study described a markedly higher protein expression level in rat RMVs than BMVs with hyperglycemia-induced downregulation to occur only in RMVs^12^. In this previous study, RMVs and BMVs were isolated by different methods which may well have affected the analysis outcome. In our study, both RMVs and BMVs were isolated in an identical manner, thus allowing direct comparison of the results from both MV compartments. Our data show that in addition to Slc2A1, the insulin-sensitive glucose transporter Slc2A4 (Glut-4) also displayed a significant enrichment in RMVs and BMVs, although at much lower levels than Slc2A1. Using immunofluorescence microscopy, Slc2A4 has previously been localized in individual neurons of different brain areas^32^ and in endothelial cells of MVs isolated from the rat hypothalamus^33^. However, our data suggest a more widespread expression in the microvasculature of the brain and similarly in the retina, at a markedly lower level in RMVs than in BMVs. The presence of Slc2A4 is considered of functional importance in regulating glucose transport into neuronal tissues during hypoglycemia^34^. Based on our results we therefore speculate that RMVs may be less responsible compared to BMVs to changes in blood glucose concentrations, which subsequently may lead to impaired glucose control in the retina.

Quite uniquely, another member of the Slc family, Slc16a1 showed a considerably higher expression level in RMVs than in BMVs. Previously, Slc16a1 has been localized to brain endothelial cells, mainly on the luminal surface^35^. This protein effects transport of lactate and pyruvate, and the high expression level found in the present study may be taken to indicate a prominent role in RMVs although the functional importance of this is not yet clear.

In recent years, several critical signaling pathways of communication between endothelial cells and pericytes such as PDGFb / PDGFRb signaling, Tie2 / Ang signaling, TGF-β signaling and Notch signaling have been described^14,16^. The communication between endothelial cells and pericytes in BMVs has been shown to contribute to the formation and maintenance of the BBB integrity^36^, and of the iBRB alike^37^. Moreover, endothelial cell—pericyte interaction is of outmost importance in the regulation of (micro)vascular development, stabilization, maturation, and remodeling as reviewed elsewhere^38^. In accord, we found many genes of these signaling pathways in the pool of common genes highly enriched in the BMVs and RMVs yielding significant enrichment scores for vasculature-related pathways. By far the highest enrichment score was for the NOTCH signaling pathway, which has been suggested to play a pivotal role in the regulation of arteriogenesis and angiogenesis^39^. Despite the agreement of BMV and RMV gene expression within the common gene pool, slight differences between these MV compartments were noted. These differences are reflected in the higher number of individual genes enriched over tissue in BMVs compared to RMVs, and they are also reflected in the distinct clustering of RMVs and BMVs seen in the PCA.

In summary, we provide for the first time comparative transcriptional profiles of rat retinal and cerebral microvasculature from the same rats. In spite of largely comparable results in the groups of genes in our focus some remarkable differences were encountered as well, first of all higher expression levels of solute carrier genes in BMVs. These differences may provide further insight into the mechanism(s) underlying the higher susceptibility of retina tissue to systemic stressors such as diabetes and arterial hypertension, and they may hopefully generate new protective approaches such as stimulation of carrier(s) in RMVs. Most resasonably,the results of the current study call for a follow-up on the protein expression level, and the lack of such data on protein is a limitation of our study. In fact, a shotgun type of proteome survey would probably best reflect our transcriptional approach. First studies in this direction have already been presented for full retina and brain tissue samples^40,41^, and similar measurements should also be applicable to the RMV and BMV compartments isolated by our novel protocol. Nevertheless, our study by increasing the knowledge of gene expression patterns in retina and brain microvascular systems might well be instrumental to understand why retina and brain show different susceptibilities to stressors and even provide new targets for specific pharmacological interventions.

## Materials and methods

### Experimental animals

This study was approved by the Animal Ethics Committee at the regional council in Karlsruhe (Germany). Application, approval, and animal care complied with the ethical regulations of the Directive 2010/63/EU, and all experimental procedures as well as data analysis and reporting met the ARRIVE guidelines as introduced by Kilkenny and coworkers^42^. Male Wistar rats purchased from Janvier (Isle St- Genest, France) were used in this study. They were housed following a standard 12-h light/dark cycle in a temperature-controlled environment, and with free access to food and tap water. Six rats (aged 12 weeks with a body weight of 546 ± 17 g) were anesthetized deeply with CO_2_ inhalation and sacrificed. Brains were dissected by removing meninges, superficial vessels, choroid plexus and white matter. Eyes and brain hemispheres were snap-frozen in liquid nitrogen and stored at -80 °C until use.

### Isolation of microvessels

The retinal and brain microvessels were isolated as described previously^13^. Briefly, frozen eyes from individual rats were cryosectioned (HBM500, Microm, Nussloch, Germany). A section of 50 µm was collected for RNA extraction from total retina tissue. The other sections were transferred into a glass tube containing 3 ml phosphate-buffered saline (PBS / 1% dextran (Dextran 70,000, Roth); PBS: NaCl 137 mM, KCl 2.7 mM, Na_2_HPO_4_ 10 mM, KH_2_PO_4_, 1.8 mM, pH 7.4). This retinal preparation was homogenized using a motor-driven homogenizer (Homgen plus, Schuett Biotec, Goettingen, Germany; 60 rpm, 20 upstrokes). For BMVs isolation, serial sections were prepared from an individual hemisphere, of which three sections were taken for total RNA extraction from full tissue. The remaining sections were homogenized in the same way as for RMVs isolation. Thereafter, the brain and retinal suspension were individually transferred onto a density gradient column (3 ml PBS / 31% dextran in the lower phase and 3 ml PBS / 18% dextran in the upper phase) and centrifuged for 15 min (1300 g). Finally, both RMVs and BMVs were captured after filtration over a 60 µm nylon mesh. All the procedures were performed at ≤ 4^o^ C.

### RNA preparations and quality control

The samples were immersed in 350 µl lysis buffer (RLT solution; Qiagen, Germany) plus 3.5 µl mercaptoethanol (Sigma), and then pulled ten times through a 22-gauge needle. Total RNA was obtained from individual MV samples or RT and BT with RNeasy® Plus Micro kit (Qiagen, Hilden, Germany) according to the manufacturer's protocol. The concentration and quantity were determined using the Agilent 6000 Pico kit (RMVs and BMVs), or Agilent Nano kit (brain and retinal tissues) on an Agilent 2100 bioanalyzer (Agilent Technologies, Waldbronn, Germany). Only samples with an integrity number (RIN) > 8.0 were used for microarray processing.

### Microarray processing

For each sample, 1 ng of total RNA was amplified using the GeneChip® WT Pico Reagent Kit (Affymetrix) according to the manufacturer's protocol. 20 μg of cRNA was used as input for the second cycle of cDNA reaction. 5.5 μg of single-stranded cDNA was used as input for the fragmentation reaction. The Affymetrix Genechip WT Terminal labeling kit was used for fragmentation and biotin labeling. Finally, the samples were hybridized to the whole-transcriptome Rat Gene 2.0 ST microarrays (Affymetrix) on the Genechip Fluidics Station 450 (Affymetrix), scanned using the Genechip Scanner 7G (Affymetrix) and the raw intensity values stored in CEL files by the GeneChip® Operating Software (Affymetrix). These raw CEL files were normalized using the Affymetrix® Expression Console Software (version 4.0, Affymetrix) and the adjusted intensity values were transformed to log2 format. The complete microarray dataset is available at Gene Expression Omnibus (GEO) database (http://www.ncbi.nlm.nih.gov/geo/) under the accession number GSE110675.

### Microarray data analysis

The microarray data were analyzed with R software using LIMMA package (version 3.02; R Development Core Team, 2013). To assess the microvascular gene expression profiles, we identified DEGs between MVs and the respective full tissue using a threshold of ≥ twofold enrichment and a FDR corrected for multiple comparisons (p < 0.05). In a second pairwise comparison, we identified DEGs between RMVs and BMVs with the same parameters applied as described above.

To investigate the functional classifications of these DEGs, biological process categories were analyzed using the Gene Ontology (GO) Consortium database (http://geneontology.org/). Pathways analysis was performed using the PANTHER Classification System^43^. Enrichment scores were obtained using a Bonferroni adjusted p < 0.05 level of significance.

### Validation of target genes by qRT-PCR

Aliquots of total RNA were taken from all samples and cDNA synthesis was performed as previously described^44^. The qRT-PCR analyses were performed in a ViiA 7 PCR System (Applied Biosystems, Nieuwerkerk aan den IJssel, The Netherlands) using the assay-on-demand primer and probe sets (from Applied Biosystems) listed in Table 1. All measurements were performed in duplicate and the mean values used for further analyses. Using the 2^-ΔCt^ methodology we calculated the ratio of the relative expression level for each of the 6 selected target genes to the expression level of 4 reference genes (for the latter the geometric mean values were used). The enrichment factors in RMVs and BMVs for each of the selected target genes were calculated for the microarray and qRT-PCR measurements (applying the 2^-ΔΔCt^ approach) and the mean values obtained from each experimental group used for correlation analysis.
